# Contamination, Source Apportionment, and Health Risk Assessment of Heavy Metals in Farmland Soils Surrounding a Typical Copper Tailings Pond

**DOI:** 10.3390/ijerph192114264

**Published:** 2022-11-01

**Authors:** Minsi Xiao, Shitong Xu, Bing Yang, Guangcong Zeng, Lidan Qian, Haiwei Huang, Sili Ren

**Affiliations:** 1 Jiangxi Key Laboratory of Mining & Metallurgy Environmental Pollution Control, Jiangxi University of Science and Technology, Ganzhou 341400, China; 2 Jiangxi Key Laboratory of Mining Engineering, Jiangxi University of Science and Technology, Ganzhou 341400, China

**Keywords:** heavy metals, farmland soils, source apportionment, APCS-MLR model, health risk assessment, mine tailings pond

## Abstract

Tailings resulting from mining and smelting activities may cause soil heavy-metal pollution and harm human health. To evaluate the environmental impact of heavy metals from tailings on farmland soils in the surrounding area, heavy metals (As, Cd, Cr, Cu, Ni, Pb, and Zn) in tailings and farmland soils in the vicinity of a typical copper tailings pond were analyzed. Contamination status, potential sources, and health risks for farmland soils were investigated. The results showed that the tailings contained a high concentration of Cu (1136.23 mg/kg). The concentrations of Cd and Cu in the farmland soils exceeded the soil quality standard. The geoaccumulation index (*I_geo_*) indicated that the soils were moderately polluted by Cu and Cd, and slightly polluted by Ni, Cr, and Zn. The absolute principal component scores–multiple linear regression (APCS-MLR) model was applied for source apportionment. The results showed that tailings release is the main source of soil heavy-metals contamination, accounting for 35.81%, followed by agricultural activities (19.41%) and traffic emission (16.31%). The health risk assessment suggested that the children in the study region were exposed to non-carcinogenic risks caused by As, while the non-carcinogenic risk to adults and the carcinogenic risk to both adults and children were at acceptable levels. It is necessary to take effective measures to control heavy-metal contamination from tailings releases to protect humans, especially children, from adverse health risks.

## 1. Introduction

The mining industry is an essential cornerstone of social construction and a key sector of the national economy [1,2,3]. Huge social and economic benefits are created in the mining process, while it creates billions of tons of tailings waste per year [4,5,6]. Massive mine tailings are generally stacked in an open tailings pond without any treatment. They occupy a large area and contain a variety of toxic heavy metals, which have potential adverse impacts on the environment and humans [7,8,9]. Previous studies have shown that tailings ponds cause heavy metals contamination of soils, dust, water, sediments, and crops in the surrounding areas [10,11,12].

Heavy metals are characterized by high toxicity, persistence, and bioaccumulation [13]. They can be existed in soils for a long time and accumulated in the food chain, which becomes a major threat to the soil environment, food security, and human health [14]. Studies have shown that soil heavy-metal contamination can cause adverse health risks to adults and children [15,16]. Investigations have been carried out on the health risks to humans exposed to heavy metals in soils contaminated by tailings in recent years, indicating soil heavy-metal pollution will enhance the risks to human health. Kamunda et al. evaluated the health risks of inhabitants exposed to heavy metals in soils from the mine tailings area and found that soil heavy-metals posed significant non-carcinogenic effects to adults and children and carcinogenic risk values were higher than acceptable values [17]. Ngole-Jeme and Fantke reported significant potential ecological and human health risks associated with metal and metalloid exposure from contaminated soils around gold mine tailings dumps [18]. Loredo-Portales et al. investigated the health risks of human exposure to mine tailings and found that non-carcinogenic and carcinogenic values of Pb and Zn in adjacent agricultural soils were above an acceptable level [19]. Furthermore, heavy-metal pollution in farmland soils may enhance the health risk of crops. Li et al. found that noncarcinogenic and carcinogenic risks of metals in Cortex Moutan near the copper tailings were higher than other sites [20]. Hao et al. found that non-carcinogenic risk and carcinogenic risk of adults and children exposed to heavy metals in farmland soils and vegetables exceeded safety levels in a lead–zinc tailings area. Therefore, it is necessary to investigate the health risks of humans exposed to heavy metals in farmland soils surrounding tailings ponds.

The contamination of heavy metals in soil is mainly influenced by natural factors (such as soil parent material) and anthropogenic activities (such as metal mining and processing, pesticide/fertilizer application, traffic, and industrial emissions) [21]. It is critical to accurately identify and quantify the sources of heavy metals in the soils for the prevention and reduction of heavy-metal contamination [22]. The specific sources of soil heavy-metal contamination can be distinguished by using source apportionment methods [23]. Recently, receptor models were widely used to quantify the source contribution of heavy metals in soils, such as positive matrix factorization (PMF), absolute principal component scores multiple linear regression (APCS-MLR), chemical mass balance (CMB), and edge analysis (UNMIX) models [24,25,26]. The APCS-MLR model can quantify the contribution of pollution sources using multivariate statistical methods, which have widely been applied in source apportionment of various contaminants in soils, dust, and sediments [27,28,29].

Copper is one of the most important metals and a significant resource for social and economic development [30]. In the past ten years, the global annual Cu excavation has steadily increased [31]. However, most of the copper mines are of low grade with a low copper concentration in China, resulting in a large number of tailings, containing toxic metals of As, Cd, Cr, Cu, Ni, Pb, Zn, etc., produced in the processes of mining and smelting [32,33]. The environmental impact of copper tailings has been largely ignored, resulting in soil pollution by heavy metals. A few studies of soil heavy-metal pollution related to copper tailings have been carried out, proving that heavy metals accumulate in soils surrounding tailings ponds [34,35]. Farmland soil quality is vital for agricultural production, food safety, and human health [36]. Farmland is also the main outdoor activity place for residents. Humans living in the tailings pond surrounds are exposed to heavy metals in farmland soil particles, and are faced with adverse health effects [37]. However, heavy-metal contamination in farmland soils surrounding copper tailings and its corresponding health risk has not been extensively investigated.

In the present study, the contamination, source apportionment, and health risk assessment of heavy metals (As, Cd, Cr, Cu, Ni, Pb, and Zn) in farmland soils surrounding a typical copper tailings pond in Jiangxi Province were investigated. The main objectives of this work are (1) to determine the contents of heavy metals in mine tailings to explore the potential pollution of the copper tailings pond, (2) to investigate the contamination status and spatial distribution of heavy metals in farmland soils, (3) to identify the potential sources of heavy metals in soils and determine the main driving factors of soil contamination, (4) to assess the health risk caused by soil heavy-metals to humans living in the area near the tailings pond. The study aims to attract people’s attention to the environmental influence of the tailings pond, provide a reference for remediating soil heavy-metal contamination from sources, and reduce health risks to humans.

## 2. Materials and Methods

### 2.1. Study Site Description and Sampling Collection

The studied copper tailings pond is in the typical non-ferrous mining area of the northeast part of Jiangxi Province in China. The predominant climate of the studied region is a subtropical monsoon climate, with an average annual temperature of 18.8 °C [38]. The mean annual precipitation of 1823 mm, with the rainy season, starts in April and ends in July. The weather is of sufficient sunshine and abundant precipitation, which is suitable for the growth of a variety of crops, one of which is rice. There is a stream near the north of the tailings pond with a total length of about 10 km. The stream flows through farmland and then falls into the river.

**Figure 1:**
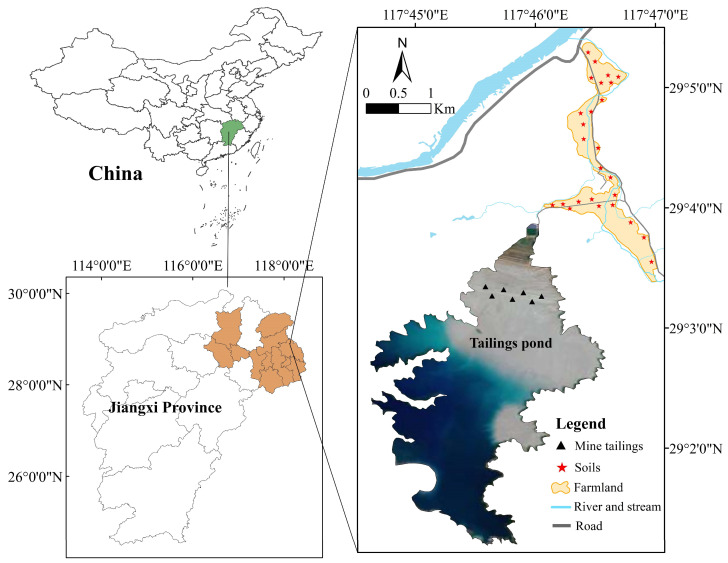
Study area and sampling sites.

The locations of sampling sites are shown in Figure 1. In the present study, 7 surface mine tailings samples (0–20 cm) and 26 surface farmland soil samples (0–20 cm) were collected. The mine tailings samples were collected from the north and south sides on the top of the tailings dam. Surface soil samples were collected from farmland in the area between the tailings pond and the river. For each sampling site of tailings and soils, five samples were mixed and reduced to appropriate weight as a composite sample by quartering method and finally put into polyethylene sealed bags. Obtained samples were transported to the laboratory for further treatment.

### 2.2. Analytical Methods

Soil and tailings samples were put on plastic film to air-dry in a cool ventilated place. Then, the dried soil and tailings samples were finely powdered in an agate mortar, and sieved with 100 mesh nylon sieves. Soil and sediment samples (approximately 0.2 g) were put into a digestion tube and digested with a 10 mL solution of 3:1:1 HNO_3_: HCl: HF (*v*/*v*) by microwave digestion system (MASTER-40, Shanghai Sineo Co., Shanghai, China). Then digestion tubes were put into the acid driver (VAVO-20, Shanghai Sineo Co., Shanghai, China) and 1 mL HClO_4_ was added periodically until the mixture was clear. After cooling down, the digested liquids were diluted by ultra-pure water to 25 mL, filtered with 0.45 μm cellulose filter, acidified with HNO_3,_ and stored in a refrigerator (4 °C). Total contents of As, Cd, Cr, Cu, Ni, and Zn were analyzed by the inductively-coupled plasma emission spectrometer (ICP-OES, PQ 9000, Analytik Jena AG Co., Jena, Germany), with the detection limit of 0.07, 0.01, 0.02, 0.04, 0.02, 0.007 ppm. Pb was analyzed by atomic absorption spectrometry (AFS, Pinaacle900F, Perkin-Elmer Co., Waltham, MA, USA), with the detection limit of 0.02 ppm. Soil pH was determined using a glass electrode method in a soil/water suspension at 1:5 ratio (Mettler-Toledo FE20, Zurich, Switzerland).

Whole program blank samples, parallel samples, and certified reference materials (GBW07388; GBW07389; National Standard Detection Research Center, Beijing, China) were used for quality control to ensure the reliability and accuracy of the analysis results. The blank and triplicate samples were carried out in the same way. The coefficients of the standard curve for these heavy metals were greater than 0.999, and the repeat sample analysis error was below 5%. The water used in the experiments was ultrapure water and reagents were guaranteed reagent (GR). The glassware was soaked in nitric acid solution (10%) for 24 h, rinsed again with pure water and dried naturally before use.

### 2.3. Contamination Assessment Based on the Geo-Accumulation Index

The geoaccumulation index (*I_geo_*) was widely used to study the heavy-metals enrichment in soils, which was calculated as follows [39]:

(1) $$I_{geo}=\log_{2}\left(C_{i}/kC_{B}\right)$$

where *C_i_* is the concentration of each heavy metal in soils (mg/kg); *k* is the corrected coefficient, which takes into account variation of background value caused by anthropogenic influences or lithologic variations in the soils (in general *k* = 1.5) [40]. *C_B_* is the geochemical background value of each heavy metal in soils (mg/kg). The soil background values of Jiangxi province were used as the reference values of *C_B_*, and the values of As, Cd, Cr, Cu, Ni, Pb, and Zn were 14.9, 0.108, 45.9, 20.3, 18.9, 32.3, and 69.4 mg/kg, respectively [41]. *I_geo_* is classified into seven levels and the corresponding contamination degrees of each heavy metal are as follows: (1) *I_geo_* ≤ 0 indicates uncontaminated; (2) 0 < *I_geo_* ≤ 1 indicates uncontaminated to moderately contaminated; (3) 1 < *I_geo_* ≤ 2 indicates moderately contaminated; (4) 2 < *I_geo_* ≤ 3 indicates moderately contaminated to heavily contaminated; (5) 3 < *I_geo_* ≤ 4 indicates heavily contaminated; (6) 4 < *I_geo_* ≤ 5 indicates heavily to extremely contaminated; (7) *I_geo_* > 5 indicates extremely contaminated.

### 2.4. Multivariate Statistical Analysis and APCS-MLR Model

In the present study, the multivariate statistical analyses were conducted in SPSS 23.0 (IBM, Inc., Armonk, NY, USA). The correlation analysis (CA) was used to characterize the strength of the relationship between the heavy metals, where the significant positive correlations among heavy metals indicate that they may share similar sources. Principal component analysis (PCA) can reveal the relationship between heavy metals and classify them by the method of dimension reduction. Potential sources of heavy metals were identified by CA and PCA analysis. The APCS-MLR model was used to obtain quantitative information for each identified source. The basic principle of APCS-MLR is to convert the principal component scores (PCS) obtained by PCA into the absolute principal component scores (APCS) of heavy-metal concentrations and use a multiple linear regression model (MLR) to quantitatively analyze the sources and determine the contribution of each principal component to heavy-metal pollution. The APCS-MLR model was described as follows [42,43]:

(2) $$C_{i}=b_{0i}+\overset{n}{\underset{p=1}{\sum }}\left(b_{pi}\times APCS_{P}\right)$$

where *C_i_* is the concentration of each heavy metal in the soils, *b*_0*i*_ is the constant term of MLR, *b_pi_* is the regression coefficient, *APCS_p_* is APCS of principal component *p*, and *b_pi_* × *APCS_p_* is the contribution of source *p*, The average of *b_pi_* × *APCS_p_* for all samples is the average of the source *p*.

### 2.5. Human Health Risk Assessment Models

A human risk assessment is a process to evaluate the probability of adverse health effects of individuals or populations exposed to contaminations. In the present study, human health risk assessment was conducted based on models proposed by the United States Environmental Protection Agency (USEPA) [44] and models in the Technical Guidelines for Risk Assessment of Contaminated Sites [45]. Typically, humans are exposed to heavy metals in the soil via three exposure routes including oral ingestion, dermal contact, and inhalation [46]. In terms of different physical characteristics and behavior habits, we divided the crowd into adults (age: above 18 years), and children (age: 0 to 18 years). The average daily dose of heavy metals in soils for adult and child were calculated as follows:

(3) $$ADD_{ing}=\frac{C_{i}\times IR_{s}\times EF\times ED}{BW\times AT}\times 10^{-6}$$

(4) $$ADD_{der}=\frac{C_{i}\times SA\times AF\times ABS\times EF\times ED}{BW\times AT}\times 10^{-6}$$

(5) $$ADD_{inh}=\frac{C_{i}\times PM_{10}\times IR_{i}\times RF\times FS\times EF\times ED}{BW\times AT}\times 10^{-6}$$

where *ADD_ing_*, *ADD_der_*, *ADD_inh_* is the average daily dose of heavy metals in soil via oral ingestion, dermal contact, and inhalation, respectively (mg/kg/day). *C_i_* is the concentration of heavy metals in soil (mg/kg); *IR_s_* is the ingestion rate of soil (mg/day); *IR_i_* is the inhalation rate (m^3^/h); *EF* is the exposure frequency, referring to the frequency with which the exposure occurs (days/year); *ED* is the exposure duration that an individual or population is exposed to the heavy metals (years); *BW* is the body weight of an individual (kg); *AT* is the averaging time, referring to the amount of time over which exposure is averaged (days); *SA* is the exposed skin surface area (cm^2^), *AF* is the skin adherence factor (mg/cm^2^/day), and *ABS* is the dermal absorption factor (unitless); *PM*_10_ is the content of inhalable particulates in ambient air (mg/m^3^); *RF* is the retention fraction of inhaled particulates in body (unitless); and *FS* is fraction of soil-borne particulates in air (unitless). The values of exposure factors used for the health risk assessment for a standard residential exposure scenario are listed in Table S1 [45,47,48,49,50].

The adverse effect of an individual or population exposed to contaminations includes non-carcinogenic and carcinogenic [51]. The non-carcinogenic risk of heavy metals can be calculated by the hazard quotient (*HQ*) and the total non-carcinogenic risks can be characterized by the hazard index (*HI*), which is calculated as follows:

(6) $$HI=\sum HQ=\sum \frac{ADD}{RfD}$$

where *ADD* is the average daily dose (mg/kg/day); *RfD* is the reference dose (mg/kg/day). If the *HQ* or *HI* ≥ 1, it indicates potential non-carcinogenic effects, while *HQ* or *HI* < 1 indicates no non-carcinogenic risks. Values of *RfD* among the health risk assessment model parameters for each heavy metal are listed in Table S2 [45,47,52,53,54,55].

The carcinogenic risk (*CR*) refers to the probability of a person suffering from cancer due to exposure to carcinogens in his lifetime, which is calculated as follows:

(7) TCR=∑CR=∑ADD×SF

where *TCR* is the sum of the carcinogenic risk (*CR*) of different heavy metals; *SF* is the carcinogenic slope factor of each metal via different exposure routes ((mg/kg/day)^−1^). Among the seven heavy metals, only As, Cd, and, Cr carry carcinogenic risk. The theoretical value of acceptable lifetime carcinogenic risk was established by the USEPA, which ranges from 1 × 10^−4^ to 1 × 10^−6^. A risk of 1 × 10^−6^ means that one person out of one million could develop cancer as a result of lifetime exposure to carcinogens. *CR* and *TCR* ≥ 1 × 10^−4^ are considered as unacceptable, *CR* and *TCR* < 1 × 10^−6^ are considered to have no significant carcinogenic effects on humans, *CR* and *TCR* lying between 1 × 10^−4^ and 1 × 10^−6^ is considered acceptable [56]. Values of *SF* among the health risk assessment model factors for all heavy metals are provided in Table S2.

## 3. Results

### 3.1. Contents of Heavy Metals in Mine Tailings

The statistical results of heavy-metal contents in mine tailings collected from seven sampling sites are shown in Table 1. The results indicate that the mine tailings still contained toxic heavy metals with different contents after copper extraction. The mean concentrations of various heavy metals were in the decreasing order of Cu (1136.23 mg/kg) > Cr (78.92 mg/kg) > Zn (43.33 mg/kg) > Pb (27.97 mg/kg) > Ni (19.01 mg/kg) > As (17.45 mg/kg) > Cd (0.41 mg/kg). The average concentrations of As, Cd, Cr, and CuNi in tailings were 1.17, 3.80, 1.72, and 55.97 times as high as the corresponding soil background values, respectively. The concentration of Cu was much higher than that of other heavy metals, which is a considerable source of heavy metals. Despite the other metals having lower concentrations than Cu, they still have the potential of migrating into surrounding soils, water, and organisms in weathering conditions [57].

The coefficient of variation (CV) indicates the variation degree of heavy-metal concentrations, which is an important parameter that reflects the uniformity of heavy-metal distribution [42]. The CV of various heavy-metal concentrations in mine tailings were ranking in the decreasing order of As (26.62%) > Ni (18.04%) > Cd (17.81%) > Cu (11.91%) > Zn (10.29%) > Cr (8.38%) > Pb (7.84%). Obviously, As had moderate variability while the other heavy metals showed low variability, indicating that the As concentration varied to a certain extent with spatial distribution, while the other heavy metals changed little.

### 3.2. Contamination Status of Heavy Metals in Farmland Soils

Soil pH ranged from 4.98 to 6.37 with a mean value of 5.57, the soils appear acidic. This was similar to the study of Teng et al. [58], which report that the pH values varied from 3.0 to 8.9 with a mean value of 5.31 in Jiangxi province. Statistical analyses of heavy-metal contents in farmland soils are shown in Table 2. The mean concentrations of Cd, Cr, Cu, Ni, and Zn were 3.98, 2.09, 4.51, 2.69, and 1.80 times as high as their corresponding background values, respectively. Particularly, the concentrations of Cd and Cu in soils significantly exceeded the Grade II specified in the environmental quality standard for soils in China. The geoaccumulation index (*I_geo_*) was further calculated to assess the contamination of heavy metals in soils. The *I_geo_* values of all heavy metals were decreased in the order of Cu (1.53) > Cd (1.24) > Ni (0.81) > Cr (0.44) > Zn (0.25) > Pb (−1.09) > As (−1.14). It is evident that soils were moderately polluted by Cu and Cd, unpolluted to moderately polluted by Ni, Cr, and Zn, and unpolluted by As and Pb. It is corresponding to the results of Chen et al. [31] that soils near the copper mines were moderately to heavily polluted with Cu and Cd.

**Figure 2:**
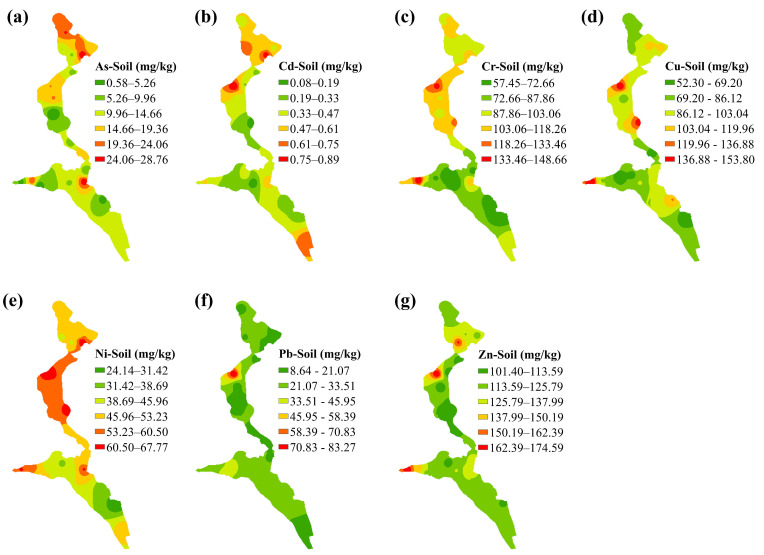
Spatial distribution of (**a**) As, (**b**) Cd, (**c**) Cr (**d**) Cu, (**e**) Ni, (**f**) Pb and (**g**) Zn in soils.

In addition, the coefficient of variation (CV) can reflect the spatial variability of heavy metals, and the low CV value indicates the influence of natural factors while the high CV value reflects the existence of anthropogenic sources [60,61]. The CV of various heavy metals concentrations were in the following decreasing order of As (59.30%) > Pb (55.93%) > Cd (47.46%) > Cu (29.35%) > Cr (24.28%) > Ni (20.40%) > Zn (15.49%) (Table 2). Combined with the spatial distribution maps, Cr, Cu, and Ni (Figure 2c–e) showed similar spatial distribution characteristics with moderate variability, the high values mainly in the central, western, and northern regions. In particular, high concentrations of Cr and Cu in soils occurred near the tailings pond, which was consistent with the high contents of Cr and Cu in tailings. It suggested that Cr, Cu, and Ni in soils may have a common source related to tailings. Moreover, dissimilar distribution trends were found in the content of As, Cd, Pb, and Zn in soils. It can be seen that As and Pb contents have high variability, indicating uneven spatial distribution. The high values of As are distributed mainly in the northern area and a few in the central and western region (Figure 2a), while the highest value of Pb is distributed in the northern area (Figure 2f), which may have resulted from anthropogenic activity. Cd showed moderate variability and its high values were distributed in the northern and southern areas (Figure 2b). The CV of Zn is very low, suggesting that typically natural factors may govern its spatial distribution. Overall, As, Cr, and Zn in the whole study region were at safe levels, while Cu exceeded the screening level. For Cr, Ni, and Pb, the percentage of samples exceeding the screening levels in the total number of samples was 73.08%, 19.23%, and 3.85%, respectively.

### 3.3. Source Identification by PCA and Source Apportionment by APCS-MLR Model

**Figure 3:**
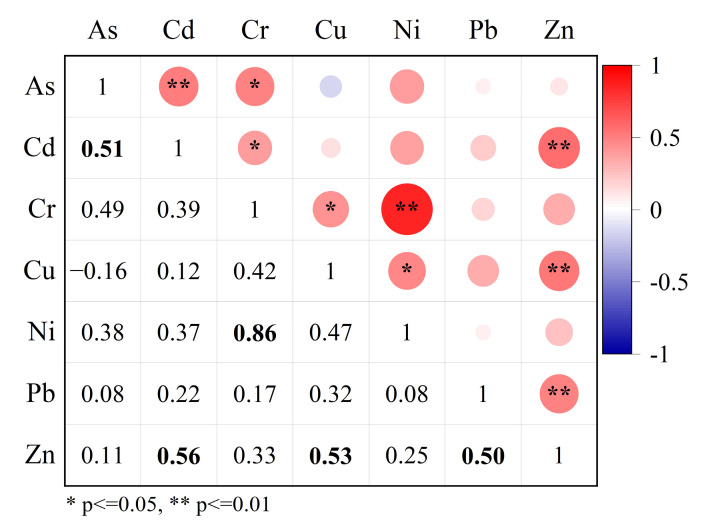
Pearson correlation analysis of heavy metals in soils. The correlation coefficients (*p* < 0.01) are remarked in bold.

Pearson correlation analysis was carried out to determine the relationship among heavy metals content in soils, and the correlation coefficients (r) are shown in Figure 3. The greater the correlation coefficient, the more likely these heavy metals have similar sources or influencing factors [27,62]. It can be seen that As-Cd, Cr-Ni, Cd-Zn, Cu-Zn, and Pb-Zn were significantly correlated at 0.01 level, while As-Cr, Cd-Cr, Cr-Cu, and Cu-Ni were significantly correlated at 0.05 level. Cr and Ni are highly correlated (r > 0.8, *p* < 0.01) while Cr-Cu and Cu-Ni are moderately correlated, indicating that Cr, Cu, and Ni may also have the same or similar sources. Zn is significantly correlated with Cd, Cu, and Pb, suggesting that there may be multiple sources. As is only significantly correlated with Cd, but not with other heavy metals, indicating that As may come from another source.

The principal component analysis (PCA) was applied to identify the sources of heavy-metal contamination in soils. The result from the KMO–Bartletts test of soil heavy-metal contents was 0.62 (Sig < 0.01), indicating that the data are strongly correlated and suitable for principal component analysis [42]. Three principal components (PCs) were extracted and explained 81.82% of the total variance of the seven heavy metals, which is sufficient to interpret the information contained in the analysis data. The factor loading matrix obtained by orthogonal rotation of the maximum variance method is shown in Table 3.

The first principal component (PC1) accounted for 30.97% of the total variation and was dominated by Cr, Cu, and Ni. The contents of Cr, Cu, and Ni in soil were higher than the corresponding background values and the results of the accumulated index also showed an obvious accumulation of these heavy metals, indicating that in addition to the geological background, there is also a source of pollution caused by human activities. Particularly, the content of Cu in soils was 4.51 times higher than the background value and the content of Cu in tailings was very high, suggesting that the increase of the content of Cu in soils was probably caused by the tailings. Combined with the spatial distribution map, Cr, Cu and Ni showed similar distribution characteristics and high concentrations of Cr and Cu occurred in the soil samplings near the tailings pond. Therefore, PC1 could be identified as the tailings release.

The second principal component (PC2) accounted for 27.30% of the total variation, which was strongly loaded in Pb and Zn. The large variation coefficient of Pb and accumulation of Zn in soils indicate that Pb and Zn were probably affected by human activities. Studies have shown that Pb mainly comes from traffic emission because of the use of catalysts and the combustion of automotive fuels [63,64], and the Zn pollution resulted from tire wear or erosion of galvanized components [60,65]. Moreover, according to the spatial distribution map (Figure 2f,g), soil samples close to the main road have higher Pb and Zn contents. Therefore, PC2 could represent the result of traffic emissions.

The third principal component (PC3) accounted for 23.54% of the total variation, which was dominated by As and Cd. As in soil showed a very high variation coefficient. The average concentration of Cd was higher than the background value with a moderate variation. These results indicated that As and Cd were probably affected by human activities. Previous studies have shown that As is the key component of pesticides, the arsenical pesticides were widely used in agricultural activities for the past many years, which may increase As concentration in soils [66,67]. In addition, a large amount of Cd exists in phosphorus fertilizer, which is a symbol element of agricultural activities [68,69]. So, PC3 could be ascribed to agricultural activities.

**Figure 4:**
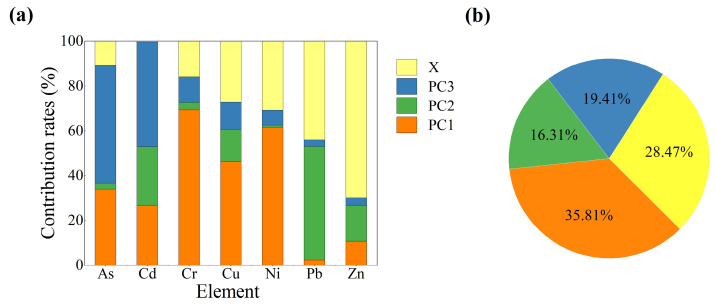
The contributions obtained from the APCS-MLR model: (**a**) Average contribution of each source to individual heavy metals in soils. (**b**) Average contribution of each source to all heavy metal content. (X represents unidentified sources).

Based on the results obtained from the PCA, the APCS-MLR model was employed to quantitatively determine the contribution of each source to heavy-metal contents in soils. Heavy-metal pollution source contribution ratios and estimated and observed data in the soil model were obtained (Table S3). The determination coefficient (R^2^) values were above 0.6 (in most cases > 0.8) and the ratios of the mean estimated values to the observed values (E/O) were close to 1 (1.00–1.02), suggesting that the source apportionment by APCS-MLR is credible and acceptable [22,70]. As shown in Figure 4a, Cr, Cu and Ni were primarily contributed by tailings release, with the contributed proportion of 69.27%, 46.21%, and 61.47%, respectively. Traffic emission was the major source of Pb, which contributed an average of 50.54% Pb concentration in soils. Agricultural activity was the main source of As and Cd, accounting for 52.45% and 46.70%, respectively. In addition, the unidentified sources also contributed a lot to heavy metals in soils, and in particular, Zn was dominated by unidentified sources. In the previous analysis, Zn is likely to be affected by natural factors due to its low coefficient of variation and contents. In summary, the APCS-MLR model well interpreted the three main sources of heavy metals including the tailings release, traffic emission, and agricultural activities, and their average contribution to the total heavy metals was 35.81%, 16.31%, and 19.41%, respectively (Figure 4b).

### 3.4. Health Risk Assessment of Heavy Metals in Farmland Soils

**Figure 5:**
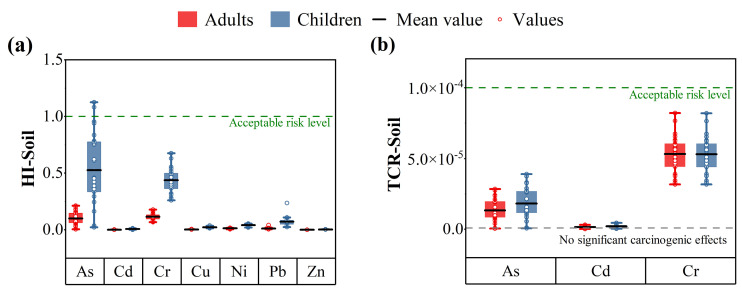
(**a**) Non-carcinogenic and (**b**) carcinogenic risks of heavy metals for children and adults.

Heavy metals can pose a potential health risk to humans through chronic exposure of polluted soil particles. The human health risks of heavy metals in soils include non-carcinogenic risk and carcinogenic risk, posed by soil heavy-metals for adults and children via three potential routes including oral ingestion, dermal contact, and inhalation [71]. The calculated results of the health risk assessment (non-carcinogenic and carcinogenic risks) of soil heavy-metals were listed in Table S4. The *HQ* and *CR* via oral ingestion are higher than dermal contact or inhalation for both adults and children in the study region. The results indicate that oral ingestion is the main exposure route that is most likely to impact health risk, which is consistent with the reports in the literatures [61,72,73]. In the present study, *HQ_ingestion_*, *HQ_dermal_*, and *HQ_inhalation_* values of seven heavy metals for children were higher than those for adults in the study region. In other words, children in the study region have higher soil non-carcinogenic risks than adults via the three exposure routes. Previous studies have also shown that children have higher risks in polluted areas due to higher soil ingestion and lower body weight [51,74,75]. It can been seen from Table S4, the non-carcinogenic risk (*HI*) values for As, Cd, Cr, Cu, Ni, Pb and Zn for adults were varied from 4.34 × 10^−3^ to 2.13 × 10^−1^, 2.95 × 10^−4^ to 3.43 × 10^−3^, 6.89 × 10^−2^ to 1.78 × 10^−1^, 2.29 × 10^−3^ to 6.73 × 10^−3^, 6.32 × 10^−3^ to 1.78 × 10^−2^, 4.38 × 10^−3^ to 4.22 × 10^−2^, and 5.95 × 10^−4^ to 1.02 × 10^−3^, while children were 2.29 × 10^−2^ to 1.13 × 10^0^, 1.14 × 10^−3^ to 1.32 × 10^−2^, 2.61 × 10^−1^ to 6.77 × 10^−1^, 1.29 × 10^−2^ to 3.79 × 10^−2^, 1.95 × 10^−2^ to 5.48 × 10^−2^, 1.28 × 10^−2^ to 2.45 × 10^−2^, and 3.34 × 10^−3^ to 5.76 × 10^−3^, respectively. According to Figure 5a, the values of *HI* of each heavy metal except As, were lower than 1, suggesting no serious non-carcinogenic health risk for both children and adults. Specifically, for children, the *HI* values of As were higher than unit (*HI* > 1), which reflects that children are exposed to adverse health effects of As in soils in the investigated region. According to the study of Teng et al., As with low *RfD* values posed a relatively higher non-carcinogenic risk to people than other trace elements [31]. The non-carcinogenic risk of each heavy metal for children was higher than that for adults, reflecting that children are more sensitive to the adverse health effects of heavy metals.

Among the studied heavy metals, As, Cd, and Cr were considered to be carcinogenic risks. It was observed from Table S4, the values of total carcinogenic risk (*TCR*) for As, Cd and Cr for adults were 5.80 × 10^−7^ to 2.85 × 10^−5^, 2.77 × 10^−7^ to 3.22 × 10^−6^, and 3.17 × 10^−5^ to 8.22 × 10^−5^, while for children the values were 7.94 × 10^−7^ to 3.91 × 10^−5^, 3.90 × 10^−7^ to 4.54 × 10^−6^, and 3.17 × 10^−5^ to 8.20 × 10^−5^, respectively. As can be seen from Figure 5b, the carcinogenic risks of heavy metals for both adults and children were within the acceptable risk range (lower than 1.00 × 10^−4^), indicating that adults and children in the study region have no significant carcinogenic risk upon exposure to the soil heavy-metals. The average *CR* of As, Cd, and Cr for adults and children conformed to the same ordering trend of Cr > As > Cd. The *CR* values of Cr for both adults and children were ranged between 10^−6^ and 10^−5^, which means that the carcinogenic risk of Cr may occur in adults and children but was acceptable.

Overall, the health risk assessment indicated oral ingestion was the main exposure route of soil heavy-metals to humans. Although no significant carcinogenic risk occurred in both children and adults in the vicinity of the tailings pond, there is a non-carcinogenic risk for children. Therefore, compared with adults, children are more vulnerable to non-carcinogenic risk and this should be paid more attention.

### 3.5. Implications of the Findings

In this work, the contamination status of heavy metals in soils surrounding a typical copper tailings pond was investigated by using rich data analysis and visualization methods, confirming that the farmland soils were polluted by heavy metals to some extent. The high contents of heavy metals in tailings indicate the potential migration of heavy metals to the surrounding environment. Combined with the actual situation of the studied area, the correlation analysis and principal component analysis determined three main sources of heavy-metal contamination in soils, including tailings release, traffic emission, and agricultural activities. The APCS-MLR model was utilized to quantify the contribution of pollution sources and further verified that tailings release is the major pollution source, proving the adverse environmental impacts of tailings on the surrounding soil. It is necessary to take effective measures, such as strict monitoring, plant restoration, and/or tailings solidification to alleviate the heavy-metal contamination in soils of the study region from the source. In the process of environmental assessment and management of a region, it is essential to take human health into account in addition to environmental quality. So, the potential risks of humans exposed to contaminated soils were also evaluated in this work. The accuracy of the health risk assessment is important for the decision-maker. Therefore, people should pay attention to improving and enriching the database of tailings reservoir areas in Jiangxi Province and even the whole country to strengthen the scientific basis of health risk assessments. Moreover, it is still not very clear whether the crops planted on the farmland soil around the tailings pond are polluted by heavy metals, so it is needed to further carry out relevant research.

## 4. Conclusions

The present study has explored the contamination status, potential sources, and health risks of heavy metals (As, Cd, Cr, Cu, Ni, Pb, and Zn) in farmland soils near a copper tailings pond. The average concentrations of As, Cd, Cr, and Cu in tailings were 1.17, 3.80, 1.72, and 55.97times as high as the corresponding soil background values, respectively. So, heavy metals in the deposited tailings have the potential to migrate and impact on the surrounding environment. The heavy-metal concentrations in the farmland soils showed that Cd and Cu in soils exceeded the soil quality standard. Average *I_geo_* values were decreased in the order of Cu (1.53) > Cd (1.24) > Ni (0.84) > Cr (0.44) > Zn (0.25) > Pb (−1.09) > As (−1.14), which reflected that the farmland soils were moderately polluted by Cd and Cu, and unpolluted to moderately polluted by Ni, Cr and Zn. The APCS-MLR model apportioned the three pollution sources of heavy metals and showed the trend of tailings release (35.81%) > agricultural activities (19.41%) > traffic emission (16.31%), indicating that tailings make a major contribution to soil heavy-metals concentrations. Moreover, human health risk assessment results showed that children were exposed to non-carcinogenic risk predominantly through As in soils, while adults were not. The carcinogenic risk caused by soil heavy-metals was acceptable for both adults and children in the study region. The main exposure route most likely to impact health risk was identified as oral ingestion. Compared with adults, children had a higher susceptibility to health risks through exposure to soil heavy-metals. The findings in this work indicated that effective measures need to be carried out to prevent heavy metals migration from tailings to soils, and protect humans, especially children, in the vicinity of copper tailings ponds from the adverse health risks caused by heavy-metal contamination in soils.

## Figures and Tables

**Table 1:** Heavy-metal contents in mine tailings (mg/kg).

	As	Cd	Cr	Cu	Ni	Pb	Zn
Mean	17.45	0.41	78.92	1136.23	19.01	27.97	43.33
Min	10.34	0.34	65.92	891.43	15.40	21.30	36.84
Max	29.88	0.60	90.55	1345.10	25.03	31.38	54.79
CV (%)	26.62	17.81	8.38	11.91	18.04	7.84	10.29

**Table 2:** Contents (mg/kg) and Igeo values of heavy metals in farmland soils.

	As	Cd	Cr	Cu	Ni	Pb	Zn
Mean	13.45	0.43	96.15	91.55	50.78	25.22	124.80
Min	0.58	0.08	57.45	52.30	24.14	8.64	101.40
Max	28.76	0.89	148.66	153.80	67.77	83.27	174.59
CV (%)	59.30	47.46	24.28	29.35	20.40	55.93	15.49
^1^ BV	14.9	0.108	45.9	20.3	18.9	32.3	69.4
^2^ Grade II	30	0.3	150	50	60	80	200
*I_geo_*	−1.14	1.24	0.44	1.53	0.81	−1.09	0.25

^1^ BV is background value for soils of Jiangxi province in China [41]. ^2^ Grade II is the environmental quality standard for soils in China [59].

**Table 3:** Rotated component matrix for soil heavy-metals.

Items	PC1	PC2	PC3
As	0.24	−0.05	**0.88**
Cd	0.18	0.47	**0.72**
Cr	**0.88**	0.12	0.34
Cu	**0.64**	0.54	−0.40
Ni	**0.93**	0.04	0.24
Pb	−0.01	**0.79**	0.04
Zn	0.21	**0.87**	0.15
Eigenvalue > 1	2.17	1.91	1.65
% of variance	30.97	27.30	23.54
% Cumulative	30.97	58.28	81.82

Significant loading factors are remarked in bold.

## Data Availability

The data presented in the present study are available upon request from the corresponding author.

## Supplementary Materials

The following supporting information can be downloaded at: https://www.mdpi.com/article/10.3390/ijerph192114264/s1, Table S1. Exposure factors used for the health risk assessment. Table S2. Reference dose (*RfD*) and carcinogenic slope factor (*SF*) of heavy metals for non-carcinogenic risk assessment. Table S3. Heavy-metal pollution source contribution ratios and estimated and observed data in soils based on APCS-MLR model. Table S4. The results of health risk assessment (non-carcinogenic and carcinogenic risks) of soil heavy-metals. References [45,47,48,49,50,52,53,54,55] are cited in the supplementary materials.
