# Supplementary material for: Contamination, Source Apportionment, and Health Risk Assessment of Heavy Metals in Farmland Soils Surrounding a Typical Copper Tailings Pond
Source: Int J Environ Res Public Health. 2022 Nov 1;19(21):14264. doi: 10.3390/ijerph192114264 (PMC9656670; doi:10.3390/ijerph192114264)
Supplement: Supplementary file 1 [file ijerph-19-14264-s001.zip › ijerph-1928740-supplementary.pdf]

Supporting Information

Contamination, Source Apportionment, and Health Risk Assessment of Heavy Metals in Farmland Soils Surrounding a Typical Copper Tailings Pond

Minsi Xiao [1], Shitong Xu [2], Bing Yang [2], Guangcong Zeng [2], Lidan Qian [2], Haiwei Huang [2] and Sili Ren [1,2,*]

[1] Jiangxi Key Laboratory of Mining & Metallurgy Environmental Pollution Control, Jiangxi University of Science and Technology, Ganzhou 341400, China
[2] Jiangxi Key Laboratory of Mining Engineering, Jiangxi University of Science and Technology, Ganzhou 341400, China
* Correspondence: sili_ren@163.com

There are 4 pages including 5 tables.

Table of Contents

Items	Pages
**Table S1.** Exposure factors used for the health risk assessment.	S3
**Table S2.** Reference dose (*RfD*) and carcinogenic slope factor (*SF*) of heavy metals for non-carcinogenic risk assessment.	S4
**Table S3.** Heavy metal pollution source contribution ratios and estimated and observed data in soils based on APCS-MLR model.	S5
**Table S4.** The results of health risk assessment (non-carcinogenic and carcinogenic risks) of soil heavy metals.	S6-S7

Table S1. Exposure factors used for the health risk assessment [1-4].

Factors	Instructions	Units	Values	
			Adults	Children
IR_s	ingestion rate of soil	mg/day	100	200
IR_i	inhalation rate	m^3/day	15.3	8.9
EF	exposure frequency	day/year	350	350
ED	exposure duration	year	24	6
BW	body weight	kg	56.0	19.7
AT	averaging time	day	70×365 for carcinogenic rick, *ED*×365 for non-carcinogenic risk	70×365 for carcinogenic rick, *ED*×365 for non-carcinogenic risk
SA	exposed skin surface area	cm^2	5700	2800
AF	skin adherence factor	mg/cm^2/day	0.07	0.2
ABS	dermal absorption factor	unitless	As:0.03; Others: 0.001	As: 0.03; Others: 0.001
PM_{10}	content of inhalable particulates in ambient air	mg/m^3	0.15	0.15
RF	retention fraction of inhaled particulates in body	unitless	0.75	0.75
FS	fraction of soil-borne particulates in air	unitless	0.5	0.5

Table S2. Reference dose (*RfD*) and carcinogenic slope factor (*SF*) of heavy metals for non-carcinogenic risk assessment [3,5-9].

Heavy metals	Ingestion RfD_{ing}	Dermal RfD_{der}	Inhalation RfD_{inh}	Ingestion SF_{ing}	Dermal SF_{der}	Inhalation SF_{inh}
As	3.00×10^{-4}	1.23×10^{-4}	3.01×10^{-4}	1.50	1.50	1.20×10
Cd	1.00×10^{-3}	1.00×10^{-5}	1.00×10^{-5}	6.10	6.10	6.30
Cr	3.00×10^{-3}	6.00×10^{-5}	2.86×10^{-5}	5.00×10^{-1}	2.00×10	4.20×10
Cu	4.00×10^{-2}	1.20×10^{-2}	4.02×10^{-2}	-	-	-
Ni	2.00×10^{-2}	5.40×10^{-4}	9.00×10^{-5}	-	-	-
Pb	3.50×10^{-3}	5.25×10^{-4}	3.52×10^{-3}	-	-	-
Zn	3.00×10^{-1}	6.00×10^{-2}	3.00×10^{-1}	-	-	-

Table S3. Heavy metal pollution source contribution ratios and estimated and observed data in soils based on APCS-MLR model.

Metals	PC1	PC2	PC3	X	Estimated value (mg/kg)	Observed value (mg/kg)	E/O	R2
As	33.90%	2.78%	**52.45%**	10.87%	13.45	13.45	1.00	0.833
Cd	26.73%	26.12%	**46.70%**	0.46%	0.44	0.43	1.02	0.767
Cr	**69.27%**	3.36%	11.43%	15.95%	96.15	96.15	1.00	0.892
Cu	**46.21%**	14.33%	12.31%	27.15%	91.55	91.55	1.00	0.867
Ni	**61.47%**	1.03%	6.67%	30.83%	50.78	50.78	1.00	0.913
Pb	2.34%	**50.54%**	3.11%	44.01%	25.22	25.22	1.00	0.632
Zn	10.75%	16.01%	3.22%	**70.02%**	124.80	124.80	1.00	0.818

Table S4. The results of health risk assessment (non-carcinogenic and carcinogenic risks) of soil heavy metals.

Elements	Groups		Non-carcinogenic risks				Carcinogenic risks			
			HQ_{ingest}	HQ_{dermal}	$HQ_{inhalation}$	HI	CR_{ingest}	CR_{dermal}	$CR_{inhalation}$	TCR
As	Adults	Min	3.34×10^{-3}	9.74×10^{-4}	2.86×10^{-5}	4.34×10^{-3}	5.15×10^{-7}	6.16×10^{-8}	3.54×10^{-9}	5.80×10^{-7}
		Max	1.64×10^{-1}	4.79×10^{-2}	1.41×10^{-3}	2.13×10^{-1}	2.53×10^{-5}	3.03×10^{-6}	1.74×10^{-7}	2.85×10^{-5}
		Mean	7.68×10^{-2}	2.24×10^{-2}	6.58×10^{-4}	9.98×10^{-2}	1.18×10^{-5}	1.42×10^{-6}	8.15×10^{-8}	1.33×10^{-5}
	Children	Min	1.90×10^{-2}	3.89×10^{-3}	4.73×10^{-5}	2.29×10^{-2}	7.31×10^{-7}	6.14×10^{-8}	1.46×10^{-9}	7.94×10^{-7}
		Max	9.33×10^{-1}	1.91×10^{-1}	2.33×10^{-3}	**1.13**	3.60×10^{-5}	3.02×10^{-6}	7.21×10^{-8}	3.91×10^{-5}
		Mean	4.36×10^{-1}	8.94×10^{-2}	1.09×10^{-3}	5.27×10^{-1}	1.68×10^{-5}	1.41×10^{-6}	3.37×10^{-8}	1.83×10^{-5}
Cd	Adults	Min	1.31×10^{-4}	5.21×10^{-5}	1.12×10^{-4}	2.95×10^{-4}	2.73×10^{-7}	1.09×10^{-9}	2.43×10^{-9}	2.77×10^{-7}
		Max	1.52×10^{-3}	6.06×10^{-4}	1.31×10^{-3}	3.43×10^{-3}	3.18×10^{-6}	1.27×10^{-8}	2.82×10^{-8}	3.22×10^{-6}
		Mean	7.44×10^{-4}	2.97×10^{-4}	6.41×10^{-4}	1.68×10^{-3}	1.56×10^{-6}	6.21×10^{-9}	1.38×10^{-8}	1.58×10^{-6}
	Children	Min	7.42×10^{-4}	2.08×10^{-4}	1.86×10^{-4}	1.14×10^{-3}	3.88×10^{-7}	1.09×10^{-9}	1.00×10^{-9}	3.90×10^{-7}
		Max	8.63×10^{-3}	2.42×10^{-3}	2.16×10^{-3}	1.32×10^{-2}	4.51×10^{-6}	1.26×10^{-8}	1.17×10^{-8}	4.54×10^{-6}
		Mean	4.23×10^{-3}	1.19×10^{-3}	1.06×10^{-3}	6.48×10^{-3}	2.21×10^{-6}	6.20×10^{-9}	5.72×10^{-9}	2.22×10^{-6}
Cr	Adults	Min	3.28×10^{-2}	6.54×10^{-3}	2.96×10^{-2}	6.89×10^{-2}	1.69×10^{-5}	2.69×10^{-6}	1.22×10^{-5}	3.17×10^{-5}
		Max	8.49×10^{-2}	1.69×10^{-2}	7.66×10^{-2}	1.78×10^{-1}	4.36×10^{-5}	6.96×10^{-6}	3.15×10^{-5}	8.22×10^{-5}
		Mean	5.49×10^{-2}	1.09×10^{-2}	4.95×10^{-2}	1.15×10^{-1}	2.82×10^{-5}	4.50×10^{-6}	2.04×10^{-5}	5.31×10^{-5}
	Children	Min	1.86×10^{-1}	2.61×10^{-2}	4.90×10^{-2}	2.61×10^{-1}	2.40×10^{-5}	2.68×10^{-6}	5.04×10^{-6}	3.17×10^{-5}
		Max	4.82×10^{-1}	6.75×10^{-2}	1.27×10^{-1}	6.77×10^{-1}	6.20×10^{-5}	6.95×10^{-6}	1.30×10^{-5}	8.20×10^{-5}
		Mean	3.12×10^{-1}	4.37×10^{-2}	8.19×10^{-2}	4.38×10^{-1}	4.01×10^{-5}	4.49×10^{-6}	8.43×10^{-6}	5.30×10^{-5}
Cu	Adults	Min	2.24×10^{-3}	2.98×10^{-5}	1.92×10^{-5}	2.29×10^{-3}	-	-	-	-
		Max	6.58×10^{-3}	8.76×10^{-5}	5.64×10^{-5}	6.73×10^{-3}	-	-	-	-
		Mean	3.92×10^{-3}	5.21×10^{-5}	3.36×10^{-5}	4.00×10^{-3}	-	-	-	-
	Children	Min	1.27×10^{-2}	1.19×10^{-4}	3.17×10^{-5}	1.29×10^{-2}	-	-	-	-
		Max	3.74×10^{-2}	3.49×10^{-4}	9.32×10^{-5}	3.79×10^{-2}	-	-	-	-
		Mean	2.23×10^{-2}	2.08×10^{-4}	5.55×10^{-5}	2.25×10^{-2}	-	-	-	-
Ni	Adults	Min	2.07×10^{-3}	3.05×10^{-4}	3.95×10^{-3}	6.32×10^{-3}	-	-	-	-
		Max	5.80×10^{-3}	8.57×10^{-4}	1.11×10^{-2}	1.78×10^{-2}	-	-	-	-
		Mean	4.35×10^{-3}	6.42×10^{-4}	8.31×10^{-3}	1.33×10^{-2}	-	-	-	-

	Children	Min	1.17×10^{-2}	1.22×10^{-3}	6.54×10^{-3}	1.95×10^{-2}	-	-	-	-
		Max	3.30×10^{-2}	3.42×10^{-3}	1.84×10^{-2}	5.48×10^{-2}	-	-	-	-
		Mean	2.47×10^{-2}	2.56×10^{-3}	1.37×10^{-2}	4.10×10^{-2}	-	-	-	-
Pb	Adults	Min	4.23×10^{-3}	1.12×10^{-4}	3.62×10^{-5}	4.38×10^{-3}	-	-	-	-
		Max	4.07×10^{-2}	1.08×10^{-3}	3.49×10^{-4}	4.22×10^{-2}	-	-	-	-
		Mean	1.23×10^{-2}	3.28×10^{-4}	1.06×10^{-4}	1.28×10^{-2}	-	-	-	-
	Children	Min	2.40×10^{-2}	4.49×10^{-4}	5.98×10^{-5}	2.45×10^{-2}	-	-	-	-
		Max	2.32×10^{-1}	4.32×10^{-3}	5.76×10^{-4}	2.37×10^{-1}	-	-	-	-
		Mean	7.01×10^{-2}	1.31×10^{-3}	1.75×10^{-4}	7.16×10^{-2}	-	-	-	-
Zn	Adults	Min	5.79×10^{-4}	1.15×10^{-5}	4.98×10^{-6}	5.95×10^{-4}	-	-	-	-
		Max	9.97×10^{-4}	1.99×10^{-5}	8.58×10^{-6}	1.02×10^{-3}	-	-	-	-
		Mean	7.12×10^{-4}	1.42×10^{-5}	6.13×10^{-6}	7.33×10^{-4}	-	-	-	-
	Children	Min	3.29×10^{-3}	4.61×10^{-5}	8.24×10^{-6}	3.34×10^{-3}	-	-	-	-
		Max	5.67×10^{-3}	7.93×10^{-5}	1.42×10^{-5}	5.76×10^{-3}	-	-	-	-
		Mean	4.05×10^{-3}	5.67×10^{-5}	1.01×10^{-5}	4.12×10^{-3}	-	-	-	-

References

1. MEPPRC. *Exposure Factors Handbook of Chinese Population (Children)*; (MEPPRC), M. o. E. P. o. t. P. s. R. o. C.: Beijing, 2016.
2. MEPPRC. *Exposure Factors Handbook of Chinese Population (Adults)*; China, M. o. E. P. o. t. P. s. R. o.: Beijing, 2013.
3. MEPC, HJ 25.3—2014 Technical guidelines for risk assessment of contaminated sites In Ministry of Environmental Protection of China, Ed. China Environmental Science Press: Beijing, 2014.
4. USEPA. *Exposure Factors Handbook: 2011 Edition EPA/600/R-09/052F*; Agency, U. S. E. P.: Washington, D.C, 2011.
5. RAIS. The Risk Assessment Information System. Available online: Available from https://rais.ornl.gov/tools/profile.php
6. Miao, F. F.; Zhang, Y. M.; Li, Y.; Fang, Q. L.; Zhou, Y. Z., Implementation of an integrated health risk assessment coupled with spatial interpolation and source contribution: a case study of soil heavy metals from an abandoned industrial area in Suzhou, China. *Stoch Env Res Risk A* **2022**, 1-15. http://10.1007/s00477-021-02146-2.
7. Kan, X.; Dong, Y.; Feng, L.; Zhou, M.; Hou, H., Contamination and health risk assessment of heavy metals in China's lead-zinc mine tailings: A meta-analysis. *Chemosphere* **2021,** *267,* 128909. http://10.1016/j.chemosphere.2020.128909.
8. Guo, G.; Wang, Y.; Zhang, D.; Li, K.; Lei, M., Human health risk apportionment from potential sources of heavy metals in agricultural soils and associated uncertainty analysis. *Environ. Geochem. Health* **2022**. http://10.1007/s10653-022-01243-7.
9. Shi, Y.; Li, Y.; Yuan, X.; Fu, J.; Ma, Q.; Wang, Q., Environmental and human health risk evaluation of heavy metals in ceramsites from municipal solid waste incineration fly ash. *Environ. Geochem. Health* **2020,** *42*, (11), 3779-3794. http://10.1007/s10653-020-00639-7.
